# Prevent Wounds by Conducting a Comprehensive Foot Examination and Intervention

**DOI:** 10.3390/healthcare3030586

**Published:** 2015-07-17

**Authors:** Michele Shelly Burdette-Taylor

**Affiliations:** Health Science Building, College of Health, School of Nursing, University of Alaska Anchorage, #357, 3211 Providence Drive, Anchorage, AK 99508, USA; E-Mail: mrburdettetaylor@uaa.alaska.edu; Tel.: +1-907-330-9928

**Keywords:** type 2 diabetes, self-care activities, adult learning principles, Certified Foot Care Nurse (CFCN), comprehensive foot exam, prevention of wounds, proactive providers, amputation prevention, chronic lower extremity wound

## Abstract

Lower extremity wounds and falls are on the rise with the demographics and projected aging population. Diabetes and heart disease supersede cancer deaths. A basic foot exam—performed routinely on patients identified as high risk allows time for early intervention and prevention. A Certified Foot and Nail Care Nurse (CFCN) who evaluates clients on a regular basis, conducts a comprehensive lower extremity exam for loss of protective sensation (LOPS) and compromised peripheral blood flow is more likely to provide needed care in a timely manner. Why a nurse? Because nurses who have the level of education, expertise through acquired training, and are board certified are competent to assess, educate, provide intervention, and refer. Utilizing CFCNs is cost-effective and efficient. CFCN is utilized as a member of the multidisciplinary team. Nurses are educators and education is an effective method for prevention. Nurses, as the most trusted health care provider, communicate, establish rapport, and develop sustaining relationships. Utilizing the Wound Ostomy Continence Nurses’ Credentialing Board (WOCNCB) CFCN raises the standard of care substantially and reduces overall costs to life, limbs, and dollars. This innovation in practice improves outcomes, patient satisfaction, and safety while reducing hospital admissions.

## 1. Introduction

Chronic costly wounds will continue to impact Medicare beneficiaries and caregivers until health care focuses on prevention by implementing proactive interventions. Pain, suffering, loss of limb, and infection are a few of the associated problems with a chronic, non-healing lower extremity wound. Eighteen million people suffer from lower extremity arterial disease (LEAD) of which many are asymptomatic [1]. Age and diabetes are two significant risk factors for LEAD. Age, smoking, and sedentary life style increase the risk of LEAD and type 2 diabetes mellitus (T2DM). Almost twenty-six million Americans suffer with diabetes of which 18.8 million are diagnosed and seven million undiagnosed. Over twenty-six percent are 65 years or older [2].

## 2. Significance

The average cost of a foot ulcer is $4,595 per episode [3]. Almost $28,000 is spent annually on each Medicare beneficiary who requires care, with a total national cost of $1.6 billion, with added costs of prior ulcer care exceeding six billion annually. Expenditures for diabetic foot patients are three times higher than the general population. Disease, disability, and related co-morbidities are devastating and expensive. Are they also avoidable?

Approximately forty-five to sixty percent of foot ulcers are due to lower extremity neuropathic disease (LEND) with the remaining forty five percent due to neuropathy and lower extremity arterial disease [1,2]. Foot ulcers and wounds are the most common reason for people with diabetes to be admitted and re-admitted to an acute care hospital. Of all foot wounds admitted, approximately seventy percent of those have no follow-up once discharged from the in-patient setting, less than two percent referred for off-loading, and eleven percent referred for home health care or a wound care center [4]. Patients’ assessment of quality or discharge instructions for care at home are strongly correlated with the overall mean score of patient satisfaction [5]. Fifty to eighty percent of all amputations are due to diabetes-related complications. Amputations are a major cause of morbidity and mortality. People with diabetes whose non-healing wounds result in amputation have a mortality rate of up to fifty percent in five years, a rate similar to several types of cancers [1,2,6].

Medicare provides beneficiaries coverage for shoes and inserts in an effort to reduce neuropathic wounds directly related to footwear. Since 1993, Medicare has provided coverage for a pair of shoes and three sets of inserts annually but only nine percent eligible are being referred for therapeutic shoes and inserts. In 2006, Medicare recognized the need for “pay for performance” for reimbursement. It is a voluntary program by reporting care delivered and referrals for the Therapeutic Shoe Bill. In 2005, the Wound Ostomy Continence Nurses’ (WOCN) Society recognized the need for a front-line nurse, to specialize in foot care for prevention of wounds [7].

Diabetes and heart disease now supersede cancer deaths in the aging population of the United States [6]. This population struggles with diminished mobility, flexibility, visual acuity, and circulation in the extremities. With the increased incidence of diabetes, and obesity—examining and caring for one’s own feet is difficult, often times impossible.

## 3. Discussion

LEAD and LEND wounds constitute a high volume of service and consumes large percentage of finite human and financial resources. There is no standardized plan of care for delivering proactive foot and nail care or an outcome based database to ascertain the efficiency of foot care practices, education, appropriate referral, procedures, and policies. A front-line service is missing—but what? A basic foot exam—performed routinely and regularly on patients identified as having the potential of developing an ulcer. [8,9,10,11]. It is imperative that foot and nail care be delivered safely, effectively, and efficiently.

The Agency for Health Care Quality and Research (AHRQ), Institute of Medicine (IOM), Healthy People 2020, and the Lower Extremity Amputation Prevention (LEAP) organizations have all recognized the necessity for early intervention that leads to prevention—of a potential complication [12,13,14,15]. Currently, there are no resources available for professionals for the high risk patient population. Though several nurse-led studies and initiatives exist they have not organized for dissemination as a universal or global initiative [8,9,16,17,18,19].

Newly enacted programs focus on preventative care and help Certified Foot and Nail Care Nurse (CFCN) assume a key role in reducing the costly impact of care for people with LEAD and LEND. A CFCN evaluates patients on a regular basis, conducts a lower extremity foot assessment specifically for loss of protective sensation (LOPS) and compromised blood flow. With frequency and deliberate surveillance there is a greater chance of discovering problems. If a pressure point is identified and the shoes worn are the culprit, the CFCN advises on appropriate off-loading and tight glucose control [15,20,21,22].

Identified barriers for patients to conduct foot self-care are physical ability, perceived importance, knowledge, education, social integration, risk status, and patient-provider communication [23]. Identified enablers for patients to conduct foot care are perceived importance, higher risk for lower extremity injury, positive patient-provider communication, experiences and relationships, strong social networks either electronic or face-to-face and education perceived as empowering and motivational [23]. By monitoring and encouraging tight glucose control and a one percent decrease in hemoglobin A1c (HbA1c) results in a forty-three percent decrease in amputation, readmission, and an eight hundred dollar reduction in health care costs per patient [24,25].

Why a nurse? Because nurses have the level of education, critical thinking, expertise, and with acquired training may become board certified in foot and nail care. They have knowledge specific to lower extremity exam and management of issues and intervention opportunities. Utilizing CFCNs would be cost-effective and efficient [6,9,26].

Certified Foot and Nail Care Nurse (CFCN) can—in 10 min or less [27,28] (Table 1).

**Table 1:** Comprehensive Foot Exam.

Exam	Instruments/Issues	Goal
Assess for Sensation	5.07 Semmes-Weinstein Monofilament128 mHz Tuning Fork	Determine loss of protective sensation and position of toe in shoe
Assess Pulses and Blood Flow	Doppler Posterior Tibialis and Dorsalis PedisConduct an Ankle-Brachial Index or Toe Pressure	Determine if compromised blood
Assess for Musculoskeletal Deformities	Hyperkeratotic lesions and balance issues	Determine pressure points, fall risk, and safety issues
Assess for Dermatologic Conditions	Malignant melanoma, Tinea Pedis, Onychomycosis	Determine and treat major / minor skin and nail conditions

Certified Foot and Nail Care Nurse (CFCN) can, based on the following assessment, qualify risk (Table 2).

**Table 2:** Risk Assessment.

Low Risk	High Risk
Low Risk (all must be present qualify low risk)	High Risk (any one qualify high risk)
Intact protective sensation	Loss of protective sensation
Adequate blood flow	Inadequate blood flow
No severe deformities	Severe deformity

Certified Foot and Nail Care Nurse (CFCN) can based on the following assessment and risk (Table 3).

**Table 3:** Foot Care Interventions/Rationale.

Interventions	Rationale
Debride toenails-reduce height and length	Safety, comfort, prevention of injuries to feet or legs
Reduce hyperkeratotic lesions	Prevention of fissures, corns, calluses, wounds, promotion of comfort
Monitor HbA1C—tight glucose control	Prevention of loss of protective sensation, wounds, falls, and amputation
Monitor foot wear and sock use—reduce edema, facilitate therapeutic shoes and inserts	Reduce wounds related to edema and pressure on feet due to ill-fitting shoes leading to wounds and amputations

Certified Foot and Nail Care Nurse (CFCN) can based on the following assessment, risk, and interventions (Table 4).

**Table 4:** Education Interventions/Rationale.

Education Interventions	Rationale
Daily inspection of feet	Prevent minor condition lead to major issue
Self-care foot care	Daily basic hygiene and moisturize, avoid bathroom or kitchen surgery
Aging related foot changes and pathology	Normal vs abnormal changes
Problems to report	Report any abnormal changes in timely manor

With implementation of a nurse-managed safety-net foot and nail care clinic, quality healthcare services can successfully be developed and implemented despite financial and staffing limitations [15]. A CFCN with the experience and expertise uses knowledge and a multidisciplinary approach to refer to specialists promptly as deemed appropriate. Delay in utilizing the team approach is the greatest reason for people with diabetes and others with compromised blood flow to lead to amputations. A seemingly minor condition such as an ingrowing or ingrown toe nail can lead to a major problem and amputation. Lack of therapeutic foot care and identifying who is at risk is compounded with falls in the same population, those with LEAD and LEND especially of the frail vulnerable older population [29,30,31,32].

## 4. Conclusions

This initiative for innovation in practice is in direct response to the AHRQ, IOM, Healthy People 2020, and LEAP initiatives, mandates, and programs. The AHRQ and IOM have charged nurses to practice to the full extent of their education and develop innovative health care proposals specifically to lead change, improve care, and reduce costs. Healthy People 2020 and LEAP challenged health care providers to address the high cost of foot ulcers and reduce the costs of lives and limbs lost and dollars spent. The CFCN is considered a front-line proactive provider in prevention, providing information and incorporating a weekly phone call to check on the beneficiary is essential for patient satisfaction [33].

Utilizing the Wound Ostomy Continence Nurses’ Credentialing Board (WOCNCB) Certified Foot and Nail Care Nurse (CFCN) raises the standard of care substantially and reduces overall costs to life, limbs, and Medicare dollars [34]. This innovation in practice to improve health care delivery includes diagnostics and intervention that focuses on early detection of lower extremity neuropathic and arterial disease. This proposal is a simple innovation that could have tremendous positive outcomes worldwide. The CFCN can be the case manager of the multidisciplinary team approach to the prevention of wounds and amputations and ensure alignment with AHRQ, IOM, Healthy People 2020, and LEAP initiatives.

## 5. Key Messages

1. Amputations, falls, and wounds are related to poor or lack of foot care, lower extremity deformities, and loss of protective sensation.
2. Lack of foot and nail care is the most neglected area of health care in every setting.
3. The two most important components of a lower extremity assessment is determining loss of protective sensation and compromised blood flow.
4. Amputations, falls, and wounds are costly in resources utilized, admissions, readmissions, and treatment.
5. A Certified Foot and Nail Care Nurse (CFCN) is a proactive provider to case manage high risk individuals, monitor glucose control and foot wear, and provide basic foot and nail care.
